# Regional frequency variation of revascularization procedures for carotid stenosis in Germany: Secondary data analysis of DRG data from 2012 to 2014

**DOI:** 10.1007/s00772-018-0415-7

**Published:** 2018-07-20

**Authors:** A. Kuehnl, M. Salvermoser, E. Knipfer, A. Zimmermann, V. Schmid, H.‑H. Eckstein

**Affiliations:** 1 Department of Vascular and Endovascular Surgery, Klinikum rechts der Isar, Technical University of Munich, Ismaninger Straße 22, 81675 Munich, Germany; 2 Department of Statistics, Ludwig-Maximilians-University Munich, Munich, Germany

**Keywords:** Carotid stenosis, Surgery incidence, Regional analysis, Secondary data analysis, Health services research, Karotisstenose, Operationshäufigkeit, Regionale Analyse, Sekundärdatenanalyse, Versorgungsforschung

## Abstract

**Background:**

For Germany, regional variation of procedure rates of carotid endarterectomy (CEA) and carotid artery stenting (CAS) performed for carotid stenosis have so far not been analyzed at a national level. The aim of this study was to assess small area estimates of procedure rates among German regions, and to identify regional characteristics, which are associated with the regional frequency of procedures.

**Methods:**

German diagnosis-related groups (DRG) statistics (2012–2014) were analyzed. Inclusion and exclusion criteria for procedural codes were set according to German quality assurance measures in combination with the diagnosis of carotid stenosis (I65.2). Rates of CEA and CAS were indirectly standardized for sex and age.

**Results:**

In total, 88,182 procedures were performed (73,042 CEA; 15,367 CAS). The overall procedure rate varied between 13.2 per 100,000 (Augsburg) and 89.2 per 100,000 (Wilhelmshaven). Spatial analysis revealed that regional distribution was significantly clustered.

**Conclusion:**

The rates of CEA, and especially of CAS showed high regional variation. The spatial distribution was significantly clustered. In addition to the regional prevalence of diabetes mellitus, smoking and obesity, socioeconomic factors, such as income and debts were correlated with the overall frequency of CEA and CAS. No significant association was found between indicators of health infrastructure (e. g. density of hospital beds, vascular surgeons and angiologists) and the overall procedure rate.

****Electronic supplementary material**:**

The online version of this article (10.1007/s00772-018-0415-7) includes further information on study limitations, as well as additional tables and figures. The article and supplementary material are available at http://www.springermedizin.de/gefaesschirurgie. The additional material can be found at the end of the article under “supplementary material”.

## Zusammenfassung

**Hintergrund:**

Die kleinräumige Häufigkeit der in Deutschland durchgeführten Karotisendarteriektomien (CEA) und Karotisstentangioplastien (CAS) wurde bisher nicht auf nationalem Niveau analysiert. Ziel dieser Studie war es, die regionale Häufigkeit von CEA- und CAS-Prozeduren bei Karotisstenose (I65.2) zu beschreiben und statistisch zu analysieren sowie regionale Charakteristika zu identifizieren, die mit der regionalen Prozedurenhäufigkeit assoziiert waren.

**Methode:**

Mittels kontrollierter Datenfernverarbeitung wurde die DRG-Statistik des Statistischen Bundesamtes (StBA) von 2012 bis 2014 ausgewertet. Die Ein- und Ausschlusskriterien entsprachen den OPS-Codes der externen Qualitätssicherung (QS-Filter). Die regionale Häufigkeit von CEA- und CAS-Prozeduren wurde indirekt alters- und geschlechtsstandardisiert.

**Ergebnisse:**

Zwischen 2012 und 2014 wurden insgesamt 88.182 Prozeduren, davon 73.042 CEA und 15.367 CAS eingeschlossen. Die alters- und geschlechtsstandardisierte Häufigkeit aller Prozeduren (CEA + CAS) schwankte zwischen 13,2 pro 100.000 Einwohnern (kreisfreie Stadt Augsburg) und 89,2 Prozeduren (kreisfreie Stadt Wilhelmshaven). Die räumlich statistische Analyse ergab für die Häufigkeit von CEA und CAS ein geclustertes Verteilungsmuster.

**Schlussfolgerung:**

Die Prozedurenhäufigkeiten von CEA und vor allem von CAS wiesen eine hohe Variation und ein geclustertes räumliches Verteilungsmuster auf. Neben der regionalen Prävalenz von Diabetes mellitus, Rauchen und Adipositas waren sozioökonomische Faktoren wie Einkommensverhältnisse und Schulden mit der Gesamtprozedurenhäufigkeit assoziiert, nicht jedoch Indikatoren der Gesundheitsinfrastruktur (u. a. Dichte von Krankenhausbetten, Gefäßchirurgen, Angiologen).

## Introduction

Every year, between 15,000 and 20,000 individuals in Germany suffer a stroke caused by arterio-arterial embolisms due to atherosclerotic plaque of the carotid artery [1]. Standard revascularization procedures such as carotid endarterectomy (CEA) and carotid artery stenting (CAS) are available for both primary and secondary prevention. Evidence-based recommendations on the diagnosis, indications, choice of procedure, and follow-up have been published in national and international guidelines [1, 2].

All CEA and CAS procedures performed in German hospitals are subject to statutory external quality assurance (eQA), which includes indicators of outcome quality and details of inpatient treatment [3]. Secondary data analyses on specific questions, such as the link between age, gender, annual center volume, surgical technique, and the perioperative stroke and death rates have already been published [4–8]. Due to data protection regulations, it was not possible to carry out hospital-specific evaluations. In addition, eQA does not record information on patients’ place of residence, rendering it essentially impossible to make a differentiated analysis in terms of location.

An analysis of the frequency of surgical procedures in selected service areas revealed a moderate variation between the 402 German districts and cities in terms of procedure frequency for appendectomies and cesarean sections standardized for age and gender [9]. In contrast, coronary interventions and pacemaker implantation showed a high systematic variation [9]. So far, the small-area frequency of CEA and CAS procedures carried out in Germany was not analyzed. Although a certain regional variation should be statistically expected and conclusions should be drawn with caution [10], regional differences in the frequency of surgical procedures can be used to generate hypotheses in order to identify determinants of suboptimal care processes (e. g., access to the health system, supplier-induced increase in demand) [11–14]. Therefore, the aim of this study was: (a) to analyze the small-area frequency of CEA and CAS procedures for carotid stenosis in Germany in terms of place of residence, and (b) to identify regional characteristics (exploratory approach) associated with the regional frequency of CEA and CAS.

## Methods

### Data source and processing

The German diagnosis-related group (G-DRG) statistics compiled by the German Federal Statistical Office (Statistische Bundesamt, StBA) for the period 2012–2014 were evaluated using controlled remote data processing (CRDP; [15]). The methods used have already been described in detail elsewhere [16, 17]. The data are stored on StBA servers in accordance with the StBa data protection regulations. Staff at the Research Data Center ensured compliance with data protection regulations. The population consisted of all patients treated in a hospital on an inpatient basis. The study, which covered the whole of Germany, was approved by the Ethics Committee at the Technical University of Munich and was conducted in accordance with current guidelines [18, 19]. The assignment of regional variables (Tables 1, 2 and 3) to the cases was carried out on a place of residence basis using the official municipality key. The case-specific DRG was used to create a link to the G‑DRG catalog. The sex and age group-specific number of inhabitants (www.genesis.destatis.de), the type of district and region in terms of residential areas (German Federal Institute for Research on Building, Urban Affairs and Spatial Development, Bundesinstitut für Bau, Stadt- und Raumforschung), data on health system infrastructure (www.inkar.de, German Association of Statutory Health Insurance Physicians, Kassenärztliche Bundesvereinigung), data on the prevalence of risk factors (www.versorgungsatlas.de, www.gbe-bund.de), the DRG casemix valuation ratio, and the average maximum length of stay were linked. The codes for identifying comorbidities, procedures, and secondary outcomes are listed in eTable 1.

**Table 1:** Characteristics of the patient cohort (aggregated 2012–2014)

	CEA		CAS		Total^a^	
Total (%)	73,042	(100)	15,367	(100)	88,182	(100)
Males (%)	49,727	(68)	10,711	(70)	60,282	(68)
Age (years, median, Q1–3)	72	[65–77]	71	[63–76]	72	[65–77]
Elixhauser score (median, Q1–3)	3	[0–8]	4	[0–9]	3	[0–8]
As principal hospital diagnosis	57,632	(79)	11,305	(74)	68,789	(78)
*Documented secondary diseases*						
CHD (I25)	20,767	(28)	5066	(33)	25,764	(29)
Other^b^ heart diseases	18,406	(25)	3957	(26)	22,295	(25)
Peripheral arterial disease	16,312	(22)	3988	(26)	20,241	(23)
Arterial hypertension	60,226	(82)	12,086	(79)	72,135	(82)
Chronic lung disease	6779	(9.3)	1195	(7.8)	7954	(9.0)
Diabetes mellitus	22,045	(30)	4521	(29)	26,497	(30)
Kidney failure	11,058	(15)	2563	(17)	13,590	(15)
Cancer	984	(1.3)	262	(1.7)	1241	(1.4)
Coagulopathy	2952	(4.0)	493	(3.2)	3434	(3.9)
Obesity	5934	(8.1)	997	(6.5)	6914	(7.8)
*District type: patient place of residence*						
City	19,263	(26)	4354	(28)	23,543	(27)
Urban district	28,634	(39)	5977	(39)	34,531	(39)
Rural district	13,180	(18)	2581	(17)	15,723	(18)
Sparsely populated district	11,965	(16)	2455	(16)	14,385	(16)
*Admission type*						
Planned admission	49,850	(68)	10,517	(68)	60,236	(68)
Emergency admission	17,442	(24)	3889	(25)	21,262	(24)
Transfer	5750	(7.9)	961	(6.3)	6684	(7.6)
*District type: hospital*						
City	34,254	(47)	8404	(55)	42,658	(48)
Urban district	20,453	(28)	3856	(25)	24,309	(27)
Rural district	8709	(12)	1488	(10)	10,197	(12)
Sparsely populated district	9626	(13)	1619	(11)	11,245	(13)
*Distance: place of residence to hospital*						
Linear distance (km, median, Q1–3)	11.0	[5.3–22]	12.3	[5.6–26]	11.1	[5.3–22.2]

Percentages relate to the column unless otherwise stated

*CEA* carotid endarterectomy, *CAS* carotid angioplasty and stenting, *CHD* coronary heart disease

^a^In the “total” column, patients that underwent CEA and CAS were counted only once

^b^Heart failure, arrhythmia, or heart valve disease

**Table 2:** Diagnosis, management, and outcome (aggregated 2012–2014)

	CEA *n* = 73,042		CAS *n* = 15,367		Total^a^ *n* = 88,182	
*Revascularization procedure*						
CEA only	72,815	(100)	–	–	72,815	(83)
CAS only	–	–	15,140	(98.5)	15,140	(17)
Combined CEA/CAS	*227*	*(0.3)*	*227*	*(1.5)*	227	(0.3)
*Annual case numbers* ^*b*^						
All (CEA, CAS)	82	[50–129]	81	[48–133]	82	[49–130]
*Diagnosis and treatment*						
CT angiography (head/neck)	12,662	(17)	2574	(17)	15,137	(17)
MR angiography (head/neck)	10,416	(14)	2149	(14)	12,520	(14)
DSA (neck vessels)	23,237	(32)	11,901	(77)	34,960	(40)
Treatment on a stroke unit^c^	1607	(2.2)	790	(5.1)	2387	(2.7)
Intensive care^d^	16,540	(23)	1478	(9.6)	17,941	(20)
Artificial respiration (yes/no)	2615	(3.6)	807	(5.3)	3385	(3.8)
*Complications (coded)*						
Hospital mortality	632	(0.9)	222	(1.4)	840	(1.0)
Acute MI (I21, I22)	625	(0.9)	136	(0.9)	753	(0.9)
Resuscitation (8–77)	509	(0.7)	80	(0.5)	584	(0.7)
*Hospital stay/DRG*						
Patient hospital stay	7	[5–10]	4	[3–9]	6	[5–10]
Case mix index	1.51	[1.47–2.00]	1.65	[1.62–2.71]	1.51	[1.47–2.25]
*Type of discharge (survivors)*						
Regular discharge home	66,000	(91)	13,376	(88)	79,215	(90)
Discharge against medical advice	368	(0.5)	144	(1.0)	510	(0.6)
Transfer to rehabilitation center (099)	3184	(4.4)	889	(5.9)	4039	(4.6)
Transfer to another hospital (079, 089)	2340	(3.2)	620	(4.1)	2945	(3.3)
Other type of discharge^e^	518	(0.7)	116	(0.8)	633	(0.7)

Percentages relate to the column unless otherwise stated

*CEA* carotid endarterectomy, *CAS* carotid artery stenting, *MI* myocardial infarction, *CT *computed tomography, *MR* magnetic resonance, *DSA* digital subtraction angiography, *DRG* diagnosis-related groups

^a^In the “total” column, patients that underwent CEA and CAS were counted only once

^b^The number of cases relates to the entire hospital (same institute identifier + same location)

^c^Stroke units in accordance with Annex 2, key 6 to the data transfer agreement § 301 (3) of the German Social Code, Book V

^d^Intensive care unit in accordance with Annex 2, key 6 to the data transfer agreement § 301 (3) of the German Social Code, Book V

^e^This includes other transfer destinations (e. g., hospice, psychiatric facilities) and administrative grounds for discharge (e. g., change of insurance provider or change of remuneration system code)

**Table 3:** Correlation between regional characteristics and procedure frequency, as well as a comparison of regions with high and low procedure frequencies

Regional characteristics (*n* = 402 districts)	Correlation Spearman’s r	Procedure frequency (CEA + CAS)				
		In the lower decile		In the upper decile		*p*-Value
*Prevalence risk factors* (in %)						
Type 2 diabetes	*0.227* ^*b*^	8.34	(7.62–9.24)	9.35	(8.30–10.41)	*0.005*
Rate of smokers	*0.128* ^*b*^	27.9	(26.8–30.4)	31.3	(27.9–32.8)	*0.008*
Obesity rate	*0.260* ^*b*^	14.9	(14.2–16.4)	18.0	(16.00–20.4)	*<0.001*
*Health system infrastructure*						
Driving time to next hospital (min)	0.039	10	(4–13)	10	(5–13)	0.885
Number of hospitals^a^	−0.089	2.13	(1.46–3.40)	1.78	(1.25–2.41)	0.080
Total number of beds^a^	0.053	384	(281–750)	454	(338–653)	0.402
Surgical beds^a^	0.079	91	(68–178)	125	(79–146)	0.361
Internal medicine beds^a^	*0.105* ^*b*^	123	(96–217)	148	(126–180)	0.441
Neurological beds^a^	−0.007	13	(0–39)	15	(0–32)	0.941
Physicians^a^	−0.046	149	(128–234)	148	(132–164)	0.482
General practitioners^a^	−0.076	64.3	(59.9–66.9)	62.8	(57.1–67.5)	0.384
Vascular surgeons (SHI physicians)^a^	0.014	0.797	(0.32–1.40)	0.606	(0.00–1.11)	0.569
Angiologists (SHI physicians)^a^	0.001	0.136	(0.00–0.70)	0.0	(0.00–0.61)	0.444
*Economic factors*						
Gross domestic product in T€ per wage earner	*−0.116* ^*b*^	61.4	(59.0–66.2)	58.4	(54.2–63.6)	*0.022*
Household income in € per inhabitant	*−0.220* ^*b*^	1807	(1682–1926)	1668	(1458–1780)	*0.002*
Size of household (persons)	−0.014	2.17	(1.98–2.25)	2.15	(2.00–2.23)	0.886
Old age poverty > 65 years (%)	−0.020	16.0	(12.3–27.1)	19.5	(10.6–28.2)	0.452
Debtors per 100 inhabitants	*0.156* ^*b*^	7.95	(6.55–9.40)	9.70	(8.30–11.50)	*0.003*
Public debt in € per inhabitant	*0.150* ^*b*^	801	(588–1513)	1475	(1027–2212)	*0.003*
Unemployment rate in %	*0.218* ^*b*^	4.30	(3.15–5.95)	6.30	(4.65–9.65)	*<0.001*
*Other factors*						
Population density (inhabitants per km^2^)	−0.063	221	(124–423)	176	(106–764)	0.518
Inward migration (per 1000 inhabitants)	*−0.239* ^*b*^	47.9	(40.8–66.8)	40.0	(29.0–45.4)	*0.001*
Outward migration (per 1000 inhabitants)	*−0.231* ^*b*^	43.3	(37.2–58.1)	36.2	(29.8–41.0)	*0.002*
Proportion of foreigners in %	−0.151	7.00	(5.15–10.25)	5.25	(2.95–10.35)	0.117
School leavers with university entrance qualification in %	0.043	29.9	(22.0–39.6)	31.2	(24.2–39.7)	0.615
Self-employed per 1000 wage earners	*−0.100* ^*b*^	114	(92–137)	110	(90–123)	0.174
Care recipients (per 10,000 inhabitants)	*0.287* ^*b*^	285	(262–340)	370	(313–400)	*<0.001*
*Type of district: place of residence*						
City	–	6	(15%)	7	(18%)	0.404^c^ 0.388^d^
Urban district	–	17	(43%)	13	(33%)	
Rural district with approaches at population densification	–	10	(25%)	7	(18%)	
Sparsely populated rural districts	–	7	(18%)	13	(33%)	
*Type of region: place of residence*						
Urban region	–	9	(23%)	12	(30%)	0.207^c^ 0.768^d^
Region with approaches at population densification	–	21	(53%)	13	(33%)	
Rural region	–	10	(25%)	15	(38%)	

The figures represent the median as well as the first and third quartile

*SHI* statutory health insurance

^a^Per 100,000 inhabitants

^b^Significant on the 5% level

^c^Fisher’s exact test

^d^Cochran-Armitage trend test

### Case definition

All DRG cases in the reporting years 2012–2014 for which CEA or CAS was coded with the German Operations and Procedures Key (Operationen- und Prozedurenschlüssel, OPS), at the same time as being coded with the principal or secondary diagnosis carotid stenosis I65.2 (International Classification of Diseases ICD-10), were included. Cases with anatomically or etiologically unspecific codes (I65.3/8/9, I63.0/2) were excluded. eFigure 1 shows the patient flow chart, while eTables 2–3 list the characteristics of excluded patients. All analyses refer to one hospital case (analysis unit). With respect to the OPS codes, inclusion and exclusion criteria correspond to the official QA filter of the eQA (module 10/2, sqg.de, eTable 4–7), with the exception of minimal adjustments in the case of a single OPS code (5396.00–.03, blood vessel transposition).

### Primary study variable and spatial resolution

The frequencies of CEA and CAS procedures (= hospital incidence, primary study variable) were calculated as indirect age and sex-standardized values [17]. All frequencies refer to patients’ place of residence (not the place of treatment) and to 100,000 inhabitants. Due to the expected low incidence at district and town level (NUTS3 Level, Nomenclature des Unités Territoriales Statistiques), the reporting years 2012–2014 were aggregated. In order to show CEA and CAS separately, it was necessary to reduce spatial resolution to spatial planning regions (so-called “Raumordnungsregionen”, http://www.bbsr.bund.de) in order to avoid blocks for data protection reasons and to strike a balance between spatial resolution, currentness of data, and statistical accuracy.

### Statistical analysis

Categorical data are given as absolute frequencies with percentages. The median, 25% and 75% quantiles (Q1, Q3) were calculated uniformly for metric variables. The 95% confidence intervals (95% CI) for ranked forest plots were calculated using the method described by Sahai and Khursid [20]. Regional variation was calculated using the systematic component of variation (SCV) and the proportion of SCV in the total variation (SCV proportion; [21–23]). Global Moran’s I was calculated to analyze spatial autocorrelation and Getis-Ord Gi* was calculated to identify hot and cold spots. Standard ArcGIS algorithms (version 10.1, Environmental Systems Research Institute, Redlands, CA, USA) were used to this end. Data processing and analysis (in the form of CRDP, using the “NewVar Macro” version 1.2 provided by the StBA) was carried out with the SAS statistical program, version 9.2, for Microsoft Windows (Copyright © 2015 SAS Institute Inc., Cary, NC, USA). The graphic representation of data was prepared using Microsoft Excel and the statistics program R (version 3.2.1; The R Foundation, https://www.r-project.org). Statistical significance was set at α = 0.05.

### Hypothesis-generating exploratory analyses

To investigate the relationship between regional characteristics (Table 3) and the frequency of CEA/CAS procedures, an exploratory (ecological study design) rank correlation analysis (Spearman) was performed on a NUTS3 level (*n* = 402 districts and towns). To enable the reader to estimate the correlations on the original scale, the raw values of NUTS3 regions with low procedure frequency (lower decile) and high procedure frequency (upper decile) were additionally compared. The choice of statistical method was guided by the empirical distribution evaluated with Q‑Q plots (t-test in the case of normal distribution, otherwise the Wilcoxon rank-sum test).

## Results

### Characteristics of patients included

A total of 88,182 procedures, 73,042 CEA and 15,367 CAS, were included in the study (Table 1) between 2012 and 2014. In 227 cases, both procedures were documented. Of the patients two thirds were male, and the median age of the total cohort was 72 years. The most common comorbidities included arterial hypertension (82%), diabetes mellitus (30%), and coronary heart disease (29%). The majority of patients lived in an urban district (39%) or a city (27%). Treatment most frequently took place in independent cities (48%), followed by urban districts (27%). eFigure 2 shows the cross tables on place of residence and place of treatment in the case of CEA and CAS. The median annual number of cases per hospital was 82 (Table 2).

Hospital mortality was 1.0%. Since the times at which a stroke, a transient ischemic attack, or other symptoms occur are not coded in DRG data, it was not possible to distinguish between index events and complications; therefore, this data was not evaluated. Patients spent a median of 6 days in hospital and were discharged home normally in 90% of cases or transferred to a rehabilitation facility in 5% of cases. The mean (arithmetic) case mix index was 1.51. Further details are provided in Tables 1 and 2.

### Regional variation

**Fig. 1:**
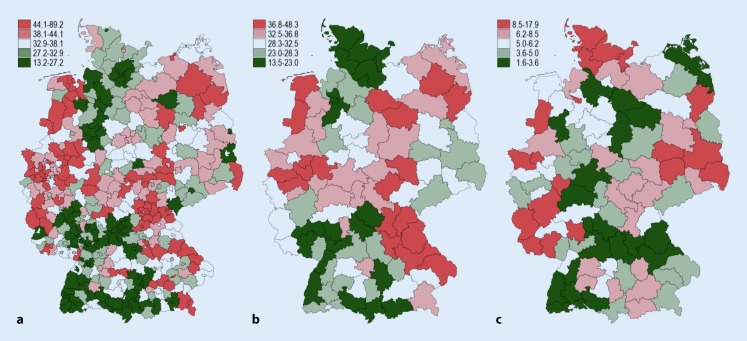
Age and sex standardized frequency of procedures per 100,000 inhabitants in the period 2012–2014. Spatial resolution refers to patients’ place of residence (districts and cities in **a**, spatial planning region in **b** and **c**). **a** Procedure frequency (CEA + CAS; global spatial autocorrelation: Moran’s I = 0.43; *p* < 0.001 clustered pattern). **b** Procedure frequency (CEA; global spatial autocorrelation: Moran’s I = 0.47; *p* < 0.001 clustered pattern). **c** Procedure frequency (CAS; global spatial autocorrelation: Moran’s I = 0.32; *p* < 0.001 clustered pattern)

**Fig. 2:**
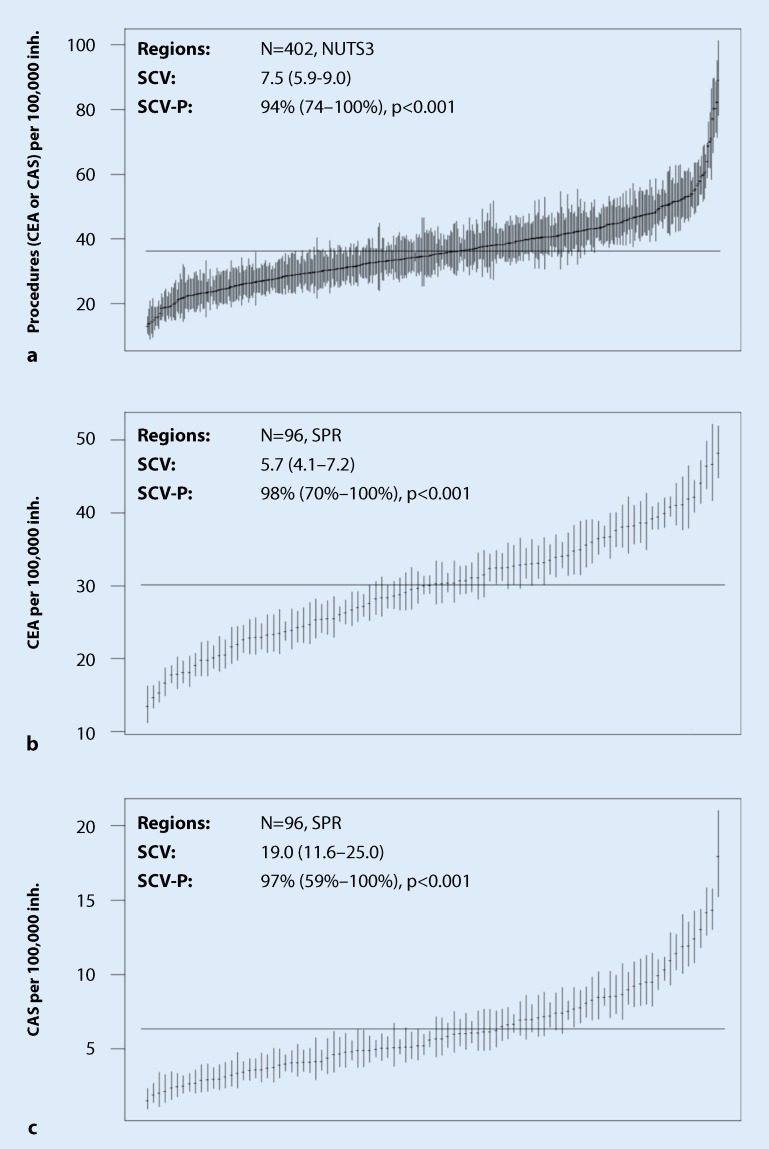
Ranked “forest plot” of age and sex standardized procedure frequency per 100,000 inhabitants (*inh.*) in the period 2012–2014. Spatial resolution refers to patients’ place of residence (NUTS3 region in **a**, spatial planning region [*SPR*] in **b** and **c**)

The overall frequency (CEA + CAS at NUTS3 level) varied between 13.2 per 100,000 inhabitants (city of Augsburg) and 89.2 procedures (city of Wilhelmshaven). The CEA frequency varied at the planning region level from 13.5 (southwest Schleswig-Holstein, Germany) to 48.3 (east Upper Franconia, Germany) CEA per 100,000 inhabitants. The CAS procedures were most rarely carried out in the Schwarzwald-Baar-Heuberg region (1.55 per 100,000 inhabitants) and most frequently in the southwest region of Schleswig-Holstein (17.9 per 100,000 inhabitants). A cartographic representation is shown in Fig. 1 and a ranked forest plot in Fig. 2. Spatial statistical analysis revealed a clustered distribution pattern for both the total frequency (CEA + CAS) and the frequency of CEA and CAS (positive global autocorrelation, *p*-value < 0.001 each). Non-random variation was 95% (SCV 7.5) for all procedures, 95% (SCV 5.7) for CEA, and 97% (SCV 19.0) for CAS.

The highest percentage of CEA procedures was found in Altmark (95.8%), while the lowest were found in southwest Schleswig-Holstein (43.1%), central Schleswig-Holstein (59.1%), and western Rhineland-Palatinate (59.4%). The distribution of the CEA percentage is shown in eFigure 3.

### Exploratory analysis (to generate hypotheses)

The regional prevalence of risk factors was significantly and positively correlated with the overall frequency of CEA and CAS, as was the concentration of internal medicine beds (Table 3). Better income levels correlated with a lower frequency of procedures and debts with a higher frequency. Fewer CEAs and CASs were carried out in regions with high levels of inward and outward migration and high numbers of self-employed individuals, while more CEAs and CASs were performed in regions with high numbers of individuals in need of care. A comparison of regions with high (upper decile) and low (lower decile) procedure frequencies revealed significant differences for the same factors (with the exception of the concentration of internal medicine beds). Neither the type of district nor type of region in terms of residential areas was significantly associated with the frequency of procedures. There was also no significant correlation with characteristics of healthcare infrastructure, such as density of beds, resident vascular surgeons or angiologists. See Table 3 for further details.

## Discussion

This study shows that the frequency of CEA and, in particular, CAS procedures for carotid stenosis in Germany is subject to significant regional variation. Spatial analysis revealed a clustered distribution pattern. In addition to the regional prevalence of diabetes mellitus, smoking, and obesity, socioeconomic factors, such as income and debts were associated with the overall frequency of procedures, but not indicators of health infrastructure.

A total of 73,000 CEA and 15,000 CAS procedures were included in the study period. When including the 7600 CEA and 4300 CAS performed in patients without specifically coded carotid stenosis (I65.2) (eTable 2, Cohort 2), these figures from the DRG statistics are consistent with eQA figures [3]. Although the two reporting channels are not independent of each other, they are subject to different control processes and incentives to document data. While it is not possible to assign the administrative DRG data to the indication groups A (asymptomatic), B (symptomatic, elective), and C (acute stroke, crescendo TIA, stroke-in-evolution, interventions under special conditions) clinically defined in eQA, it can be assumed that the majority of patients included in this study can be assigned to elective procedures. The higher mortality rate (4% vs. 1%) and higher Elixhauser comorbidity score (7 vs. 3) in the excluded patients (eTable 2, Cohort 2) points to this; however, due to different coding practices (e. g., crescendo TIA) or absent or non-specific administrative codes, it is not possible to rule out vagueness in the distinction between cohorts. Restricting patients to those that had both carotid stenosis and a matching procedure code results in a smaller but more clearly defined cohort and better reflects the elective care processes that do not overlap with emergency procedures.

Secondary data analysis of eQA data showed that nearly two thirds of CAS patients were classified in American Society of Anaesthesiology (ASA) categories I and II, while CEA patients were classified with ASA III in over two thirds of cases [3]. The latter is in line with the authors’ experience, whereas the former appears counterintuitive, particularly with respect to guideline recommendations [1]. In this study, however, the median Elixhauser comorbidity score was slightly higher for CAS compared with CEA (4 vs. 3). Although it is not possible to make a direct comparison between clinical evaluation with ASA categories and the administrative Elixhauser comorbidity score, a systematic underestimation of ASA category in CAS in the context of eQA seems probable. This should be the subject of critical discussion at the German Institute for Quality Assurance and Transparency in Healthcare (Institut für Qualitätssicherung und Transparenz im Gesundheitswesen, IQTiG) and the regional authorities for quality assurance, since ASA category is used for risk adjustment. Likewise, a consideration of relevant risk factors, such as hypertension, pre-existing cardiovascular diseases, or statin therapy, should be discussed [1].

The frequency of CEA varied between 14 and 48 per 100,000 residents (extremal coefficient 3.6). This value is comparatively low compared with the USA, for which a 9- to 10-fold variation between states has been described; it corresponds approximately to the variation in Canada [12–14]. The SCV for CEA was 5.7, which can be interpreted as a high variation [22] and lies (for comparison purposes) between that of cesarean sections (2.3), appendectomies (3.9), and percutaneous coronary interventions (7.8; [9]). In comparison, CAS has an extremely high variation with an SCV of 19. The fluctuation range (1.6–18 per 100,000 inhabitants) with an extremal coefficient of 12 is also higher than, e. g., in the USA (6–7; [13, 14]). The most recent study on regional distribution in the USA described an extremal coefficient of 8.6 for CEA and 13.9 for CAS, whereby these values relate to the Medicare population only [24].

Although standardization minimized sex and age effects in the present study, some variation still remained. Amongst others, this may be explained by differences in the prevalence of unobserved factors. The overall frequency of procedures was particularly high in, e. g., northern Bavaria, Mecklenburg-Western Pomerania, and North Rhine-Westphalia. A possible explanation for this might be the varying distribution of prevalence of risk factors, such as diabetes mellitus, smoking, or obesity, which could only be correlated on a regionally aggregated level (ecological study design), and the significant positive correlations identified (Table 3) must be interpreted with caution. As Anderson concluded, variation in the utilization of processes is not necessarily a bad thing per se [10] and can be the result of, e. g., unobserved factors.

The significant variation seen for CAS and the statistical outliers (3 out of 96 spatial planning regions) in the CAS share (eFigure 3) are, in the authors’ view, not only due to unobserved confounders, but presumably also due to differing indications; however, in the light of current guidelines, it is not possible to evaluate indication practices, particularly with administrative data. Different indication practices alone are probably not able to explain the spatially clustered distribution patterns, which, for instance, show a high frequency for CEA and a low frequency for CAS in north-eastern Bavaria and the opposite in Schleswig-Holstein, since the distance between the place of residence and treatment was less than 22 km linear distance for 75% of patients (Table 1).

An analysis of spatial variation could be used to generate hypotheses, e. g., using geocoded eQA data, to analyze where, how, and why carotid stenosis was treated—and with what outcome [10, 11]. It must be borne in mind that conservatively treated patients have not been covered as yet by eQA and only rarely by DRG data. Therefore, conclusions on the accuracy of the indication cannot be drawn with the current eQA procedure.

A positive correlation between smoking prevalence and CEA procedures was also observed in the USA [25]. Economic indicators such as debts were also positive, whereas household income correlated negatively with the frequency of procedures in Germany. The higher the affluence in a region, the fewer CEA and CAS procedures were carried out. A similar correlation has been described in the USA [25] and is consistent with other studies showing a positive link between high socioeconomic status and low cardiovascular morbidity [26].

In contrast, no correlation was seen between the frequency of CEA and CAS and parameters of health system infrastructure, such as the density of hospitals, general practitioners, vascular surgeons or angiologists. This is consistent with studies in the USA, which also found no correlation between the density of vascular surgeons and CEA frequency [25]. In that particular study, a link was found between a high concentration of beds and CEA frequency, which, in the German data was shown only for the density of internal medicine beds. Ultimately, however, there is no evidence of inadequate vascular health care services in Germany.

In summary, the study shows the extent of heterogeneity both in the indications and in the choice of method to treat carotid stenosis in Germany. Although natural variation and actual differences in incidence are likely, these cannot fully explain the observed variation. This raises the question of the extent to which national S3 guidelines on carotid stenosis have been adopted and the social question on nationwide, demand-oriented treatment for everyone.

Particularly with respect to the German Government’s “quality offensive” and the IQTiG’s efforts to improve eQA, this analysis, which in the authors’ opinion captures the most important, practice-relevant quintessence of the issue, raises the question of whether non-consideration of regional aspects and separating eQA analyses according to CEA and CAS represent a relevant weakness in the quality assurance process. Therefore, the inclusion of regional parameters in the evaluation of eQA data should be examined in order to assess outcome quality from the perspective of individual service providers, and not separately from the quality of indications, choice of procedure, and patient selection from the point of view of the regional patient population.

### Limitations

The basic limitations of the data and methods used for this analysis have already been described [17, 27] and are discussed in more detail in the online supplement (eMethods). The most relevant limitations are the following:

- The data are not findings made in the clinical setting, but administrative claims data for the purposes of hospital reimbursement.
- Since clinical details, e. g., the degree of stenosis or initial neurological symptoms, are not coded in the DRG data, no conclusions can be drawn on the quality of the indications, choice of procedure or guideline conformity.
- The StBA’s DRG statistics do not document which diagnoses were already present on admission, making it impossible to reliably differentiate between comorbidity and complication; similarly, it was not possible to measure neurological outcome.
- The follow-up period covered only inpatient stays.
- All analyses refer to patients’ place of residence; an analysis of the place of treatment on the level of NUTS3 or regional policy region was not possible for data protection reasons.
- Exploratory analyses were performed to generate hypotheses and could only be carried out on an aggregated level. Therefore, it is not possible to rule out an ecological fallacy.

## Practical conclusion

- The total number of CEA and CAS procedures in the DRG statistics showed good agreement with eQA data. The fact that, although the two reporting channels are subject to different control processes yet are not independent of each other, points to complete data collection by eQA.
- In relation to districts and towns, the overall age and gender-standardized incidence of CEA and CAS in carotid stenosis varied between 13 and 89 per 100,000 inhabitants.
- The regional frequency of all CEA and CAS procedures demonstrated a positive spatial autocorrelation and, thus a clustered spatial pattern of distribution. The CEA and CAS were frequently performed in northern Bavaria, Mecklenburg-Western Pomerania, and North Rhine-Westphalia (44–89 per 100,000 inhabitants), whereas they were carried out less frequently in Baden-Württemberg, eastern Lower Saxony, and Schleswig-Holstein (13–27 per 100,000 inhabitants).
- Only patients from the western Rhineland-Palatinate and central and south-west Schleswig-Holstein showed high percentages of CAS (41–57%), while other regions showed CAS percentages of between 4% and 33%.
- This study shows the level of heterogeneity in both the indication and the choice of method to treat carotid stenosis in Germany. Although natural variation and differences in actual incidence are likely, they are unable to fully account for the variation observed. This raises the question of the extent to which the German S3 guidelines on carotid stenosis have been adopted, as well as the social question of nationwide, demand-oriented treatment for everyone.
- Particularly in view of the German government’s quality offensive and the IQTiG’s efforts to improve eQA this article, which captures the quintessential, practice-relevant points, raises the question as to whether non-consideration of regional aspects and separating eQS analyses according to CEA and CAS represent a relevant weakness in the quality assurance procedure. Therefore, the inclusion of regional parameters and relevant risk factors in the evaluation of eQA data should be examined in order to assess outcome quality from the perspective of the individual service providers, and not separately from the quality of patient selection, indication, and choice of procedure from the point of view of the regional patient population.

## Caption Electronic Supplementary Material

Supplementary information on methodological limitations, additional figures and tables, and technical specifications.
