# Supplementary material for: Regional frequency variation of revascularization procedures for carotid stenosis in Germany: Secondary data analysis of DRG data from 2012 to 2014
Source: Gefasschirurgie. 2018 Jul 20;23(Suppl 2):56–65. doi: 10.1007/s00772-018-0415-7 (PMC6096714; doi:10.1007/s00772-018-0415-7)
Supplement: Supplementary file 1 — Supplementary information on methodological limitations, additional figures and tables, and technical specifications. [file 772_2018_415_MOESM1_ESM.pdf]

## eMethods: limitations

Due to its design and methodology, this study has a number of limitations:

- The data are not findings and complications recorded in a clinical setting, but administrative claims data. Since the documentation was used for the purposes of hospital reimbursement, differences and changes in local coding quality and policy may have led to distortions. However, one can assume with regard to the main study variables (diagnosis of carotid stenosis and subsequent CEA or CAS) that the usual controls carried out by the medical service of the German statutory health insurance (*Medizinischer Dienst der Krankenversicherung*, MDK) minimized relevant miscoding. However, it is likely that secondary diagnoses that were not relevant for remuneration were documented less frequently, and thus the analysis incorrectly reports excessively low frequencies.
- Since the StBA's DRG statistics do not document which diagnoses already existed at the time of admission, it was not possible to distinguish between the indication for the intervention (e.g., symptomatic carotid stenosis) and possible complications arising therefrom (e.g., transitory ischemic attack following the intervention) for some events. Thus, despite age and gender standardization, a certain level of bias is possible due to regional differences in the actual (yet latent) prevalence of carotid stenosis in the population and the frequency of its detection (screening, symptoms, etc.).
- Cases not coded for carotid stenosis (I65.2) were excluded; however, in the authors' opinion, it is likely that some of the patients in the excluded cohort 2 should be assigned to indication group C (CEA/CAS under particular conditions). Thus, it should be borne in mind that the conclusions drawn by this study refer primarily to CEA and CAS (indication groups A and B in eQA) presumably performed on an elective basis. Since data collected for eQA do not contain any information on patients' place of residence, a similar analysis using quality assurance data is essentially not possible, nor is a secondary data analysis according to non-anonymous hospital locations possible.
- Since the follow-up period covered only inpatient stay, complications that occurred following hospital discharge were not recorded. Any relevant bias with regard to the frequency of revascularization procedures is unlikely.
- All analyses refer to patients' place of residence. For data protection reasons, an analysis of the treatment location would only have been possible at federal state level and thus would therefore not have been reliable. As such, one cannot necessarily infer the place of treatment from the place of residence. However, since this study focuses on the patient perspective (the treatment received by patients in a particular region), this is of secondary relevance.

– Exploratory analyses could only be carried out on an aggregated level (ecological study design). Since the variables examined are characteristics of the regions and not of the individuals (regional frequency of CEA/CAS, physician density, population density, type of residential area, etc.), it is not possible to exclude an ecological fallacy. The exploratory approach was deliberately designed as a broad search for factors associated with the frequency of surgical procedures and served to generate hypotheses.

## eFigures:

eFig. 1. Patient flow diagram

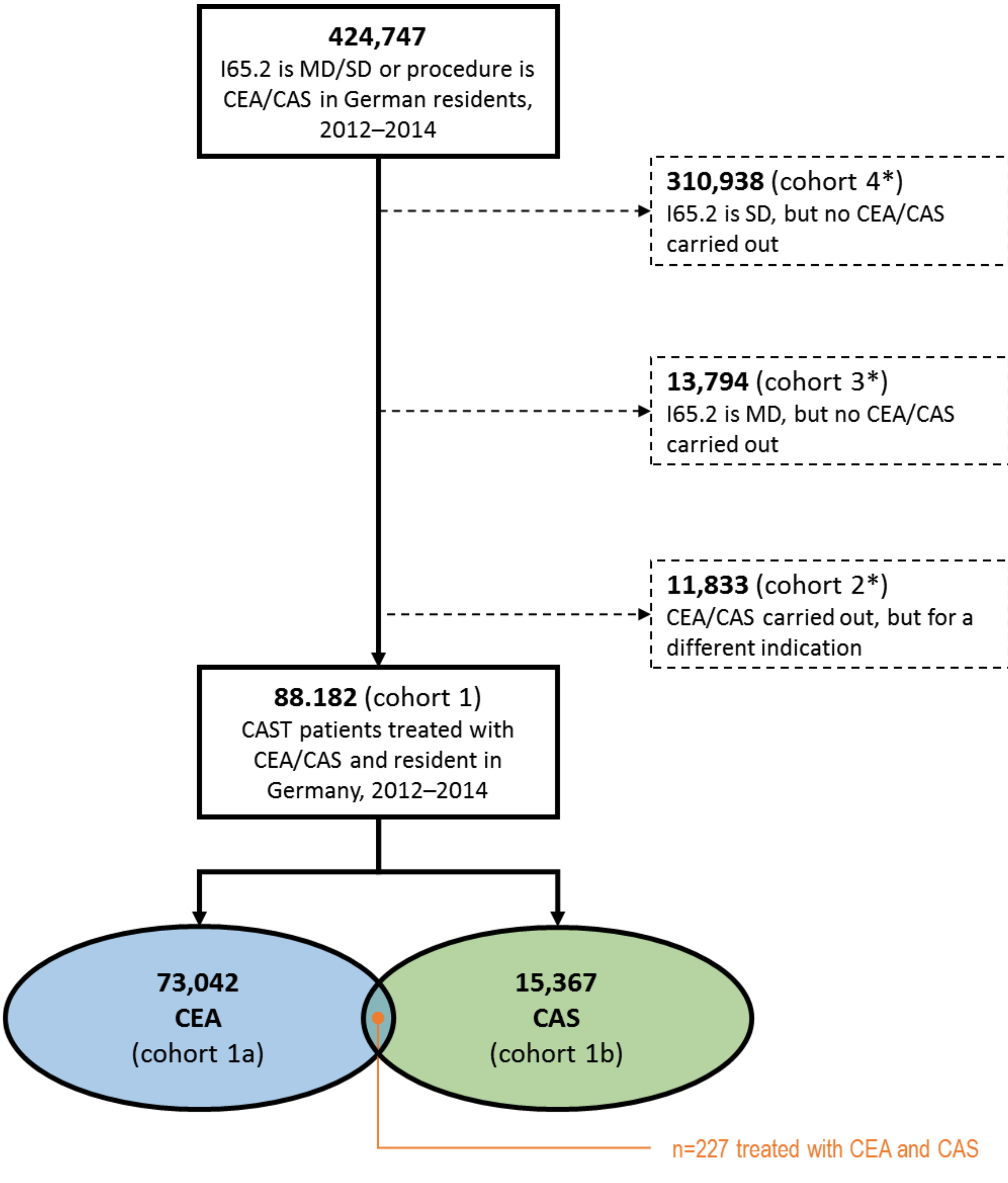


*MD*, main diagnosis; *SD*, secondary diagnosis; *CAST*, carotid artery stenosis; *CEA*, carotid endarterectomy; *CAS*, carotid stent angioplasty; *, the characteristics of excluded cohorts can be found in eTable 2 and 3

eFig. 2. Absolute case numbers according to place of residence and treatment

**CEA**

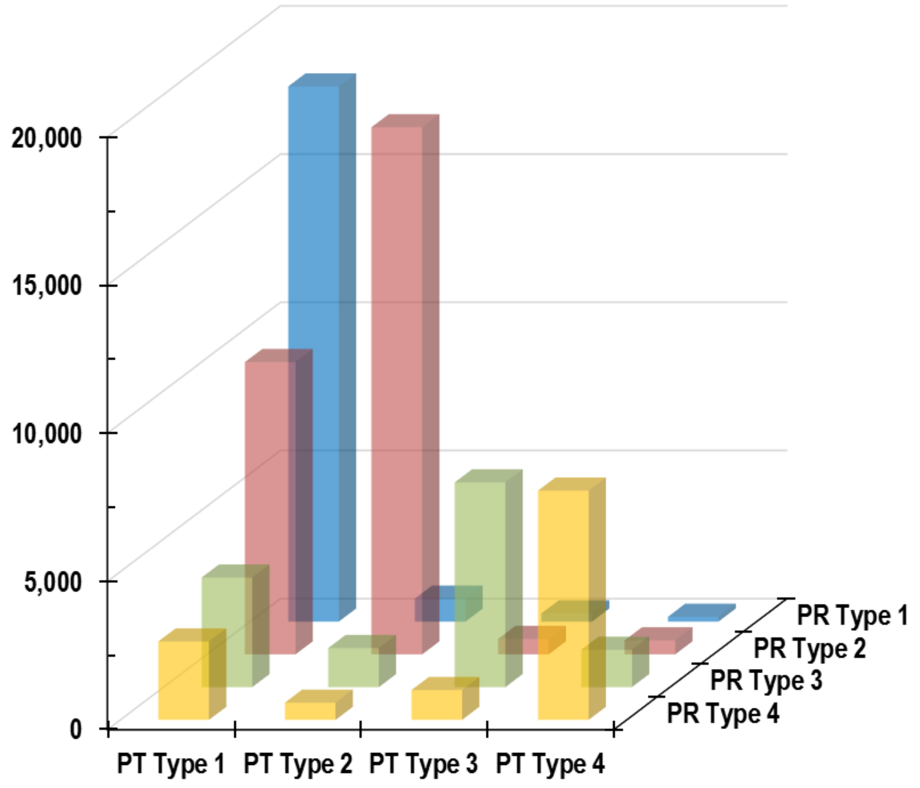


**CAS**

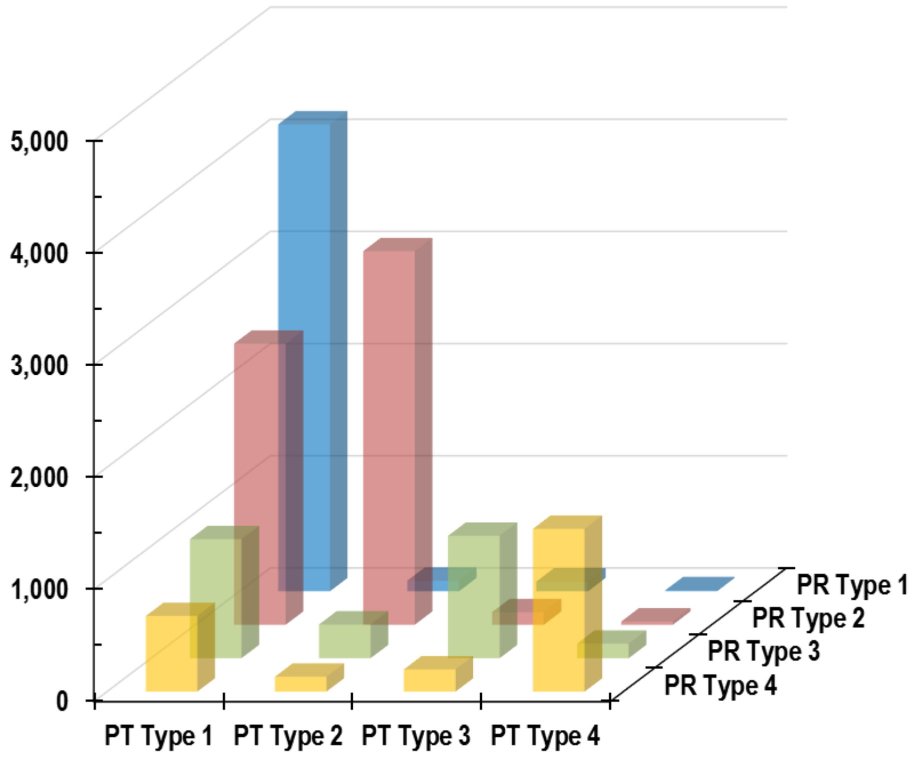


*PR*, place of residence, *PT*, place of treatment, *CEA*, carotid endarterectomy; *CAS*, carotid stent angioplasty; *Type 1*, independent city; *Type 2*, urban district; *Type 3*, rural district; *Type 4*, sparsely populated district

eFig. 3. Percentage share of CEA of all procedures shown as a ranked forest plot (**A**) and according to frequency (**B**)

A)

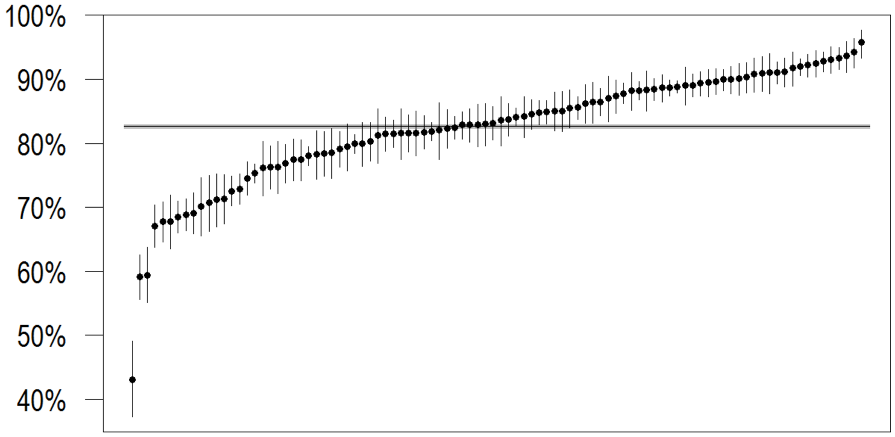


B)

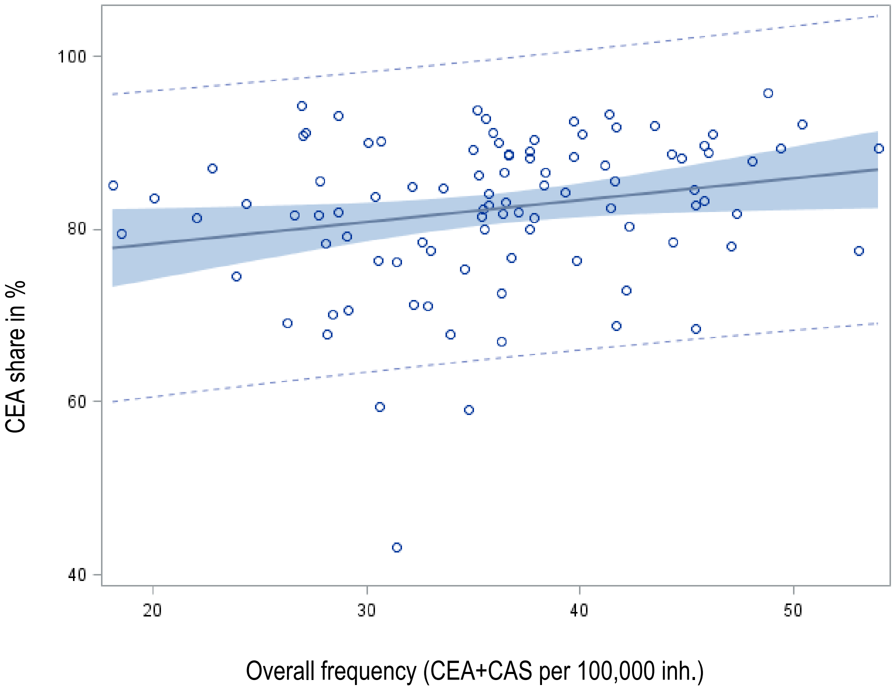


**Percentage of CEA of all procedures**

| Rank | GPR | in % | Rank | GPR | in % |
|---|---|---|---|---|---|
| 1. | Altmark | 95.8 | 92. | Northern Schleswig-Holstein | 67.8 |
| 2. | Schwarzwald-Baar-Heuberg | 94.2 | 93. | Lausitz-Spreewald | 67.0 |
| 3. | Bavarian Lower Main | 93.7 | 94. | Western Palatinate | 59.4 |
| 4. | Regensburg | 93.4 | 95. | Central Schleswig-Holstein | 59.1 |
| 5. | Upper Rhine-Lake Constance | 93.1 | 96. | South West Schleswig-Holstein | 43.1 |

*CEA*, carotid endarterectomy, *GPR*, German planning region, *inh*, inhabitants. *Dashes lines*, 95% prediction interval; *band*, 95% confidence interval.

## eTables:

eTable 1. The codes used from the German Operations and Procedures Key (*Operationen- und Prozedurenschlüssel*, OPS) and the ICD-10-GM catalog (www.dimdi.de).

| Variable | Source | Code, reference |
|---|---|---|
| Carotid stenosis | ICD-10 | I65.2 |
| Carotid endarterectomy | OPS | See eTable 4 |
| Carotid stent angioplasty | OPS | See eTable 5–7 |
| ***Characteristics and comorbidities*** | | |
| Elixhauser score (ES) | Literatur | van Walraven et al. (e1), Quan et al. (e2) |
| CHD | ICD-10 | I25* |
| Other heart disease | ES (e2) | Elixhauser item No. 1, 2, and 3 |
| Cerebrovascular disease | CCI (e3) | CCI Item No. 4 |
| PAOD | ES (e2) | Elixhauser item No. 5 |
| Hypertension (arterial) | ES (e2) | Elixhauser item No. 6 |
| Chronic lung disease | ES (e2) | Elixhauser item No. 9 |
| Diabetes mellitus | ES (e2) | Elixhauser item No. 10 and 11 |
| Kidney failure | ES (e2) | Elixhauser item No. 13 |
| Cancer | ES (e2) | Elixhauser item No. 17, 18, and 19 |
| Coagulopathy | ES (e2) | Elixhauser item No. 21 |
| Obesity | ES (e2) | Elixhauser item No. 22 |
| ***Diagnosis and treatment*** | | |
| Diagnostic procedures | OPS | 1* |
| Imaging procedures | OPS | 3* |
| - CT angiography | OPS | 3-220, 3-221 |
| - MR angiography | OPS | 3-820, 3-821 |
| - Digital subtraction angiography | OPS | 3-600, 3-601 |
| Surgical procedure | OPS | 5* |
| Non-surgical procedure | OPS | 8* |
| Treatment on a stroke unit | §301 | 0156, 2856 |
| Treatment on an ICU | §301 | 3600, 3690, 3691, 3692, 3693, 3694, 3695, 3696, 3697, 3698, 3699, 0436, 1536, 2036, 2050, 2136, 2150, 3601, 3603, 3617, 3618, 3621, 3650, 3651, 3652 |
| ***Complications and secondary outcomes*** | | |
| Acute myocardial infarction | ICD-10 | I21.*, I22.* |
| Resuscitation | OPS | 8-77* |
| Acute stroke | ICD-10 | I64, I63.0-5 |
| Transient ischemic attack | ICD-10 | G45.1/2/8/9 |
| Amaurosis fugax | ICD-10 | G45.3 |

*Wildcard; ICD, International Classification of Diseases; §301, in accordance with Annex 2, Key 6 to the Data Transfer Agreement §301 (3) of the German Social Code, Book V.

eTable 2. Characteristics of excluded patients (aggregated 2012–2014)

| | Cohort 2 "Other indication" | | Cohort 3 "Diagnosis" | | Cohort 4 "Coincidence" | |
|---|---|---|---|---|---|---|
| **OPS code for CEA/CAS*** | Yes | | Not available | | Not available | |
| **ICD-10 code for carotid stenosis (I65.2)** | Not available | | Is MD | | Is SD | |
| **Total** (%) | 11,833 | (3.5) | 13,794 | (4.1) | 310,938 | (92) |
| **Males** | 8036 | (68) | 8563 | (62) | 185,700 | (60) |
| **Age** (MW, SD) | 71 | [62–77] | 73 | [64–79] | 76 | [69–82] |
| **Elixhauser total score** | 7 | [2–13] | 3 | [0–8] | 7 | [2–14] |
| **District type: place of residence** | | | | | | |
| Independent city | 3191 | (27) | 3197 | (23) | 83757 | (27) |
| Urban district | 4835 | (41) | 5106 | (37) | 121,498 | (39) |
| Rural district | 1819 | (15) | 2748 | (20) | 58571 | (19) |
| Sparsely populated district | 1988 | (17) | 2743 | (20) | 47112 | (15) |
| **Admission type** | | | | | | |
| Planned admission | 4327 | (37) | 8106 | (59) | 129825 | (42) |
| Emergency admission | 5816 | (49) | 4632 | (34) | 153742 | (49) |
| Transfer | 1690 | (14) | 1056 | (8) | 27371 | (9) |
| **District type: hospital** | | | | | | |
| Independent city | 6282 | (53) | 5879 | (43) | 124734 | (40) |
| Urban district | 3092 | (26) | 3680 | (27) | 98160 | (32) |
| Rural district | 924 | (8) | 2039 | (15) | 48458 | (16) |
| Sparsely populated district | 1535 | (13) | 2196 | (16) | 39586 | (13) |
| **Procedure (suprastructure OPS)** | | | | | | |
| - Diagnostic procedure | 3385 | (29) | 2549 | (18) | 124202 | (40) |
| - Imaging procedure | 10484 | (89) | 11190 | (81) | 177754 | (57) |
| - Surgical procedure | 8250 | (70) | 755 | (5) | 83915 | (27) |
| CEA | 7616 | (64) | – | – | – | – |
| CAS | 4266 | (36) | – | – | – | – |
| - Non-surgical procedure | 10739 | (91) | 3704 | (27) | 178780 | (57) |
| **Complications/outcome** | | | | | | |
| Hospital mortality | 469 | (4.0) | 159 | (1.2) | 11461 | (3.7) |
| Acute MI (I21, I22) | 168 | (1.4) | 81 | (0.6) | 4650 | (1.5) |
| Resuscitation (8-77*) | 186 | (1.6) | 41 | (0.3) | 3195 | (1.0) |
| Acute stroke (I64, I63.0-5) | 1084 | (9.2) | 450 | (3.3) | 5755 | (1.9) |
| **Hospital stay/DRG** | | | | | | |
| Patient hospital stay | 10 | [6–15] | 3 | [1–7] | 8 | [4–13] |
| Case mix index | 2.78 | [1.51–3.78] | 0.70 | [0.69–0.71] | 1.03 | [0.70–1.97] |
| **Type of discharge (survivors)** | | | | | | |
| Treatment completed normally | 8331 | (73) | 11333 | (83) | 237516 | (79) |
| Discharge against medical advice | 71 | (0.6) | 216 | (1.6) | 3243 | (1.1) |
| Transfer to rehabilitation center (099) | 1671 | (15) | 194 | (1.4) | 20388 | (6.8) |
| Transfer to another hospital (079, 089) | 1110 | (9.8) | 1644 | (12) | 26452 | (8.8) |
| Other type of discharge | 181 | (1.6) | 248 | (1.8) | 11878 | (4.0) |

*MD*, main diagnosis; *SD*, secondary diagnosis. *, See eTable 4–10.

eTable 3. Continuation of eTable 2

| | Cohort 2 "Other indications" | Cohort 3 "Diagnosis" | Cohort 4 "Coincidence" |
|---|---|---|---|
| **OPS code for CEA/CAS*** | Yes | Not documented | Not documented |
| **ICD-10 code for carotid stenosis (I65.2)** | Not documented | Is MD | Is SD |
| **Main diagnosis (ICD suprastructure)** | | | |
| A | 3 (0.0) | – – | 5144 (1.7) |
| B | – – | – – | 919 (0.3) |
| C | 99 (0.8) | – – | 15361 (4.9) |
| D | 12 (0.1) | – – | 4606 (1.5) |
| E | 31 (0.3) | – – | 10755 (3.5) |
| F | – – | – – | 2628 (0.8) |
| G | 224 (1.9) | – – | 26120 (8.4) |
| H | 12 (0.1) | – – | 7197 (2.3) |
| I | 11192 (94.6)° | 13794 (100.0) | 149802 (48.2) |
| J | 12 (0.1) | – – | 13157 (4.2) |
| K | 24 (0.2) | – – | 15042 (4.8) |
| L | 3 (0.0) | – – | 1664 (0.5) |
| M | 31 (0.3) | – – | 14736 (4.7) |
| N | 9 (0.1) | – – | 8360 (2.7) |
| O | – – | – – | 17 (0.0) |
| P | § – | – – | § – |
| Q | 7 (0.1) | – – | 214 (0.1) |
| R | 14 (0.1) | – – | 17116 (5.5) |
| S | 53 (0.4) | – – | 11699 (3.8) |
| T | 106 (0.9) | – – | 5359 (1.7) |
| Z | § – | – – | § – |
| **Specific secondary diseases** | | | |
| Effects of cerebral infarction (I69.3) | 499 (4.2) | 1139 (8.3) | 25374 (8.2) |
| CHD (I25*) | 2707 (23) | 3659 (27) | 122736 (39) |
| Other* heart disease (Elix 1,2,3) | 3226 (27) | 3887 (28) | 151058 (49) |
| Cerebrovascular disease (CCI 4) | 4032 (34) | 3746 (27) | 310938 (100) |
| PAOD (Elix 5) | 2587 (22) | 2816 (20) | 80155 (26) |
| Arterial hypertension (Elix 6) | 9237 (78) | 10254 (74) | 235627 (76) |
| Chronic lung disease (Elix 9) | 1025 (8.7) | 1165 (8.4) | 40052 (12.9) |
| Diabetes mellitus (Elix 10,11) | 3272 (28) | 4134 (30) | 100870 (32) |
| Kidney failure (Elix 13) | 1788 (15) | 2569 (19) | 87605 (28) |
| Cancer (Elix 17,18,19) | 261 (2.2) | 206 (1.5) | 16039 (5.2) |
| Coagulopathy (Elix 21) | 920 (7.8) | 237 (1.7) | 18538 (6.0) |
| Obesity (Elix 22) | 767 (6.5) | 732 (5.3) | 24011 (7.7) |

*See eTable 4–7; §, blocked due to data protection regulations. °, Of these, 596 (5%) were coded as I65.3/8/9 (occlusion and stenosis of several, bilateral, other, or unspecified precerebral arteries), 104 cases (0.9%) with I63.0 (cerebral infarction due to thrombosis of the basilar, carotid, or vertebral artery) and 966 cases (8.6%) with I63/64 (cerebral infarction due to thrombosis, embolism, or unspecified occlusion or stenosis of precerebral and cerebral arteries).

eTable 4. Specifications for carotid endarterectomy (CEA) according to technical specifications (quality assurance filter) of the aQua Institute, Göttingen, Germany, www.sqg.de

| | 2009 | 2010 | 2011 | 2012 | 2013 | 2014 |
|---|---|---|---|---|---|---|
| **CEA inclusion criteria** | 5-381.00 | 5-381.00 | 5-381.00 | 5-381.00 | 5-381.00 | 5-381.00 |
| | 5-381.01 | 5-381.01 | 5-381.01 | 5-381.01 | 5-381.01 | 5-381.01 |
| | 5-381.02 | 5-381.02 | 5-381.02 | 5-381.02 | 5-381.02 | 5-381.02 |
| | 5-381.03 | 5-381.03 | 5-381.03 | 5-381.03 | 5-381.03 | 5-381.03 |
| | * | 5-381.06 | 5-381.06 | 5-381.06 | 5-381.06 | 5-381.06 |
| | 5-382.00 | 5-382.00 | 5-382.00 | 5-382.00 | 5-382.00 | 5-382.00 |
| | 5-382.01 | 5-382.01 | 5-382.01 | 5-382.01 | 5-382.01 | 5-382.01 |
| | 5-382.02 | 5-382.02 | 5-382.02 | 5-382.02 | 5-382.02 | 5-382.02 |
| | 5-382.03 | 5-382.03 | 5-382.03 | 5-382.03 | 5-382.03 | 5-382.03 |
| | 5-383.00 | 5-383.00 | 5-383.00 | 5-383.00 | 5-383.00 | 5-383.00 |
| | 5-383.01 | 5-383.01 | 5-383.01 | 5-383.01 | 5-383.01 | 5-383.01 |
| | 5-383.02 | 5-383.02 | 5-383.02 | 5-383.02 | 5-383.02 | 5-383.02 |
| | 5-383.03 | 5-383.03 | 5-383.03 | 5-383.03 | 5-383.03 | 5-383.03 |
| | 5-393.00 | 5-393.00 | 5-393.00 | 5-393.00 | 5-393.00 | 5-393.00 |
| | 5-393.01 | 5-393.01 | 5-393.01 | 5-393.01 | 5-393.01 | 5-393.01 |
| | 5-395.00 | 5-395.00 | 5-395.00 | 5-395.00 | 5-395.00 | 5-395.00 |
| | 5-395.01 | 5-395.01 | 5-395.01 | 5-395.01 | 5-395.01 | 5-395.01 |
| | 5-395.02 | 5-395.02 | 5-395.02 | 5-395.02 | 5-395.02 | 5-395.02 |
| | 5-395.03 | 5-395.03 | 5-395.03 | 5-395.03 | 5-395.03 | 5-395.03 |
| | 5-396.00 | 5-396.00 | 5-396.00 | 5-396.00 | 5-396.00 | 5-396.00 |
| | 5-396.01 | 5-396.01 | 5-396.01 | 5-396.01 | 5-396.01 | 5-396.01 |
| | 5-396.02 | 5-396.02 | 5-396.02 | 5-396.02 | 5-396.02 | 5-396.02 |
| | 5-396.03 | 5-396.03 | 5-396.03 | 5-396.03 | 5-396.03 | 5-396.03 |
| | 5-397.00 | 5-397.00 | 5-397.00 | 5-397.00 | 5-397.00 | 5-397.00 |
| | 5-397.01 | 5-397.01 | 5-397.01 | 5-397.01 | 5-397.01 | 5-397.01 |
| | 5-397.02 | 5-397.02 | 5-397.02 | 5-397.02 | 5-397.02 | 5-397.02 |
| | 5-397.03 | 5-397.03 | 5-397.03 | 5-397.03 | 5-397.03 | 5-397.03 |
| **CEA exclusion criteria** | 5-393.02 | 5-393.02 | 5-393.02 | 5-393.02 | 5-393.02 | 5-393.02 |
| | 5-393.03 | 5-393.03 | 5-393.03 | 5-393.03 | 5-393.03 | 5-393.03 |
| | # | # | # | * | ~~5-396.00~~ | ~~5-396.00~~ |
| | # | # | # | * | ~~5-396.01~~ | ~~5-396.01~~ |
| | # | # | # | * | ~~5-396.02~~ | ~~5-396.02~~ |
| | # | # | # | * | ~~5-396.03~~ | ~~5-396.03~~ |
| | 5-981 | 5-981 | 5-981 | 5-981 | 5-981 | 5-981 |
| | 5-982.1 | 5-982.1 | 5-982.1 | 5-982.1 | 5-982.1 | 5-982.1 |
| | 5-982.2 | 5-982.2 | 5-982.2 | 5-982.2 | 5-982.2 | 5-982.2 |
| | 5-982.x | 5-982.x | 5-982.x | 5-982.x | 5-982.x | 5-982.x |
| | 5-982.y | 5-982.y | 5-982.y | 5-982.y | 5-982.y | 5-982.y |

The quality assurance filters of the Aqua Institute have also been for the years 2009–2011 for comparison purposes.* Code not included in QS filters; # code listed for inclusion criteria; *green*, code was added to the inclusion criteria for this study; *red*, code deleted from the exclusion criteria for this study.

eTable 5. Specifications for angioplasty/stent angioplasty according to technical specifications (quality assurance filter) of the aQua Institute, Göttingen, Germany, www.sqg.de

| | 2009 | 2010 | 2011 | 2012 | 2013 | 2014 |
|---|---|---|---|---|---|---|
| CAS inclusion criteria | 8-836.0h | 8-836.0h | 8-836.0h | 8-836.0h | 8-836.0h | 8-836.0h |
| | 8-836.0j | 8-836.0j | 8-836.0j | 8-836.0j | 8-836.0j | 8-836.0j |
| | 8-836.0k | 8-836.0k | 8-836.0k | 8-836.0k | 8-836.0k | 8-836.0k |
| | 8-836.0m | 8-836.0m | 8-836.0m | 8-836.0m | 8-836.0m | 8-836.0m |
| | 8-836.0n | 8-836.0n | 8-836.0n | 8-836.0n | 8-836.0n | 8-836.0n |
| | 8-840.0h | 8-840.0h | 8-840.0h | 8-840.0h | 8-840.0h | 8-840.0h |
| | 8-840.0j | 8-840.0j | 8-840.0j | 8-840.0j | 8-840.0j | 8-840.0j |
| | 8-840.0k | 8-840.0k | 8-840.0k | 8-840.0k | 8-840.0k | 8-840.0k |
| | 8-840.0m | 8-840.0m | 8-840.0m | 8-840.0m | 8-840.0m | 8-840.0m |
| | 8-840.0n | 8-840.0n | 8-840.0n | 8-840.0n | 8-840.0n | 8-840.0n |
| | 8-840.1h | 8-840.1h | 8-840.1h | 8-840.1h | 8-840.1h | 8-840.1h |
| | 8-840.1j | 8-840.1j | 8-840.1j | 8-840.1j | 8-840.1j | 8-840.1j |
| | 8-840.1k | 8-840.1k | 8-840.1k | 8-840.1k | 8-840.1k | 8-840.1k |
| | 8-840.1m | 8-840.1m | 8-840.1m | 8-840.1m | 8-840.1m | 8-840.1m |
| | 8-840.1n | 8-840.1n | 8-840.1n | 8-840.1n | 8-840.1n | 8-840.1n |
| | 8-841.0h | 8-841.0h | 8-841.0h | 8-841.0h | 8-841.0h | 8-841.0h |
| | 8-841.0j | 8-841.0j | 8-841.0j | 8-841.0j | 8-841.0j | 8-841.0j |
| | 8-841.0k | 8-841.0k | 8-841.0k | 8-841.0k | 8-841.0k | 8-841.0k |
| | 8-841.0m | 8-841.0m | 8-841.0m | 8-841.0m | 8-841.0m | 8-841.0m |
| | 8-841.0n | 8-841.0n | 8-841.0n | 8-841.0n | 8-841.0n | 8-841.0n |
| | 8-841.1h | 8-841.1h | 8-841.1h | 8-841.1h | 8-841.1h | 8-841.1h |
| | 8-841.1j | 8-841.1j | 8-841.1j | 8-841.1j | 8-841.1j | 8-841.1j |
| | 8-841.1k | 8-841.1k | 8-841.1k | 8-841.1k | 8-841.1k | 8-841.1k |
| | 8-841.1m | 8-841.1m | 8-841.1m | 8-841.1m | 8-841.1m | 8-841.1m |
| | 8-841.1n | 8-841.1n | 8-841.1n | 8-841.1n | 8-841.1n | 8-841.1n |
| | 8-842.0h | 8-842.0h | 8-842.0h | 8-842.0h | 8-842.0h | 8-842.0h |
| | 8-842.0j | 8-842.0j | 8-842.0j | 8-842.0j | 8-842.0j | 8-842.0j |
| | 8-842.0k | 8-842.0k | 8-842.0k | 8-842.0k | 8-842.0k | 8-842.0k |
| | 8-842.0m | 8-842.0m | 8-842.0m | 8-842.0m | 8-842.0m | 8-842.0m |
| | 8-842.0n | 8-842.0n | 8-842.0n | 8-842.0n | 8-842.0n | 8-842.0n |
| | 8-842.1h | 8-842.1h | 8-842.1h | 8-842.1h | 8-842.1h | 8-842.1h |
| | 8-842.1j | 8-842.1j | 8-842.1j | 8-842.1j | 8-842.1j | 8-842.1j |
| | 8-842.1k | 8-842.1k | 8-842.1k | 8-842.1k | 8-842.1k | 8-842.1k |
| | 8-842.1m | 8-842.1m | 8-842.1m | 8-842.1m | 8-842.1m | 8-842.1m |
| | 8-842.1n | 8-842.1n | 8-842.1n | 8-842.1n | 8-842.1n | 8-842.1n |
| | 8-843.0h | 8-843.0h | 8-843.0h | 8-843.0h | 8-843.0h | 8-843.0h |
| | 8-843.0j | 8-843.0j | 8-843.0j | 8-843.0j | 8-843.0j | 8-843.0j |
| | 8-843.0k | 8-843.0k | 8-843.0k | 8-843.0k | 8-843.0k | 8-843.0k |
| | 8-843.0m | 8-843.0m | 8-843.0m | 8-843.0m | 8-843.0m | 8-843.0m |
| | 8-843.0n | 8-843.0n | 8-843.0n | 8-843.0n | 8-843.0n | 8-843.0n |
| | 8-843.1h | 8-843.1h | 8-843.1h | 8-843.1h | 8-843.1h | 8-843.1h |
| | 8-843.1j | 8-843.1j | 8-843.1j | 8-843.1j | 8-843.1j | 8-843.1j |
| | 8-843.1k | 8-843.1k | 8-843.1k | 8-843.1k | 8-843.1k | 8-843.1k |
| | 8-843.1m | 8-843.1m | 8-843.1m | 8-843.1m | 8-843.1m | 8-843.1m |
| | 8-843.1n | 8-843.1n | 8-843.1n | 8-843.1n | 8-843.1n | 8-843.1n |
| | 8-845.0h | 8-845.0h | 8-845.0h | 8-845.0h | 8-845.0h | 8-845.0h |
| | 8-845.0j | 8-845.0j | 8-845.0j | 8-845.0j | 8-845.0j | 8-845.0j |

eTable 6. Continuation of eTable 5

| | | | | | | |
|---|---|---|---|---|---|---|
| **CAS inclusion criteria** | 8-845.0k | 8-845.0k | 8-845.0k | 8-845.0k | 8-845.0k | 8-845.0k |
| | 8-845.0m | 8-845.0m | 8-845.0m | 8-845.0m | 8-845.0m | 8-845.0m |
| | 8-845.0n | 8-845.0n | 8-845.0n | 8-845.0n | 8-845.0n | 8-845.0n |
| | 8-845.1h | 8-845.1h | 8-845.1h | 8-845.1h | 8-845.1h | 8-845.1h |
| | 8-845.1j | 8-845.1j | 8-845.1j | 8-845.1j | 8-845.1j | 8-845.1j |
| | 8-845.1k | 8-845.1k | 8-845.1k | 8-845.1k | 8-845.1k | 8-845.1k |
| | 8-845.1m | 8-845.1m | 8-845.1m | 8-845.1m | 8-845.1m | 8-845.1m |
| | 8-845.1n | 8-845.1n | 8-845.1n | 8-845.1n | 8-845.1n | 8-845.1n |
| | 8-846.0h | 8-846.0h | 8-846.0h | 8-846.0h | 8-846.0h | 8-846.0h |
| | 8-846.0j | 8-846.0j | 8-846.0j | 8-846.0j | 8-846.0j | 8-846.0j |
| | 8-846.0k | 8-846.0k | 8-846.0k | 8-846.0k | 8-846.0k | 8-846.0k |
| | 8-846.0m | 8-846.0m | 8-846.0m | 8-846.0m | 8-846.0m | 8-846.0m |
| | 8-846.0n | 8-846.0n | 8-846.0n | 8-846.0n | 8-846.0n | 8-846.0n |
| | 8-846.1h | 8-846.1h | 8-846.1h | 8-846.1h | 8-846.1h | 8-846.1h |
| | 8-846.1j | 8-846.1j | 8-846.1j | 8-846.1j | 8-846.1j | 8-846.1j |
| | 8-846.1k | 8-846.1k | 8-846.1k | 8-846.1k | 8-846.1k | 8-846.1k |
| | 8-846.1m | 8-846.1m | 8-846.1m | 8-846.1m | 8-846.1m | 8-846.1m |
| | 8-846.1n | 8-846.1n | 8-846.1n | 8-846.1n | 8-846.1n | 8-846.1n |
| | 8-848.0h | 8-848.0h | 8-848.0h | 8-848.0h | 8-848.0h | 8-848.0h |
| | 8-848.0j | 8-848.0j | 8-848.0j | 8-848.0j | 8-848.0j | 8-848.0j |
| | 8-848.0k | 8-848.0k | 8-848.0k | 8-848.0k | 8-848.0k | 8-848.0k |
| | 8-848.0m | 8-848.0m | 8-848.0m | 8-848.0m | 8-848.0m | 8-848.0m |
| | 8-848.0n | 8-848.0n | 8-848.0n | 8-848.0n | 8-848.0n | 8-848.0n |
| | 8-848.1h | 8-848.1h | 8-848.1h | 8-848.1h | 8-848.1h | 8-848.1h |
| | 8-848.1j | 8-848.1j | 8-848.1j | 8-848.1j | 8-848.1j | 8-848.1j |
| | 8-848.1k | 8-848.1k | 8-848.1k | 8-848.1k | 8-848.1k | 8-848.1k |
| | 8-848.1m | 8-848.1m | 8-848.1m | 8-848.1m | 8-848.1m | 8-848.1m |
| | 8-848.1n | 8-848.1n | 8-848.1n | 8-848.1n | 8-848.1n | 8-848.1n |
| | 8-849.0h | 8-849.0h | 8-849.0h | 8-849.0h | 8-849.0h | 8-849.0h |
| | 8-849.0j | 8-849.0j | 8-849.0j | 8-849.0j | 8-849.0j | 8-849.0j |
| | 8-849.0k | 8-849.0k | 8-849.0k | 8-849.0k | 8-849.0k | 8-849.0k |
| | 8-849.0m | 8-849.0m | 8-849.0m | 8-849.0m | 8-849.0m | 8-849.0m |
| | 8-849.0n | 8-849.0n | 8-849.0n | 8-849.0n | 8-849.0n | 8-849.0n |
| | 8-849.1h | 8-849.1h | 8-849.1h | 8-849.1h | 8-849.1h | 8-849.1h |
| | 8-849.1j | 8-849.1j | 8-849.1j | 8-849.1j | 8-849.1j | 8-849.1j |
| | 8-849.1k | 8-849.1k | 8-849.1k | 8-849.1k | 8-849.1k | 8-849.1k |
| | 8-849.1m | 8-849.1m | 8-849.1m | 8-849.1m | 8-849.1m | 8-849.1m |
| | 8-849.1n | 8-849.1n | 8-849.1n | 8-849.1n | 8-849.1n | 8-849.1n |
| | 8-84a.0h | 8-84a.0h | 8-84a.0h | 8-84a.0h | 8-84a.0h | 8-84a.0h |
| | 8-84a.0j | 8-84a.0j | 8-84a.0j | 8-84a.0j | 8-84a.0j | 8-84a.0j |
| | 8-84a.0k | 8-84a.0k | 8-84a.0k | 8-84a.0k | 8-84a.0k | 8-84a.0k |
| | 8-84a.0m | 8-84a.0m | 8-84a.0m | 8-84a.0m | 8-84a.0m | 8-84a.0m |
| | 8-84a.0n | 8-84a.0n | 8-84a.0n | 8-84a.0n | 8-84a.0n | 8-84a.0n |
| | 8-84a.1h | 8-84a.1h | 8-84a.1h | 8-84a.1h | 8-84a.1h | 8-84a.1h |
| | 8-84a.1j | 8-84a.1j | 8-84a.1j | 8-84a.1j | 8-84a.1j | 8-84a.1j |
| | 8-84a.1k | 8-84a.1k | 8-84a.1k | 8-84a.1k | 8-84a.1k | 8-84a.1k |
| | 8-84a.1m | 8-84a.1m | 8-84a.1m | 8-84a.1m | 8-84a.1m | 8-84a.1m |
| | 8-84a.1n | 8-84a.1n | 8-84a.1n | 8-84a.1n | 8-84a.1n | 8-84a.1n |

eTable 7. Continuation of eTable 6

| | | | | | | |
|---|---|---|---|---|---|---|
| **CAS exclusion criteria** | 8-852.00 | 8-852.00 | 8-852.00 | 8-852.00 | 8-852.00 | 8-852.00 |
| | 8-852.01 | 8-852.01 | 8-852.01 | 8-852.01 | 8-852.01 | 8-852.01 |
| | 8-852.03 | 8-852.03 | 8-852.03 | 8-852.03 | 8-852.03 | 8-852.03 |
| | 8-852.04 | 8-852.04 | 8-852.04 | 8-852.04 | 8-852.04 | 8-852.04 |
| | 8-852.05 | 8-852.05 | 8-852.05 | 8-852.05 | 8-852.05 | 8-852.05 |
| | 8-852.06 | 8-852.06 | 8-852.06 | 8-852.06 | 8-852.06 | 8-852.06 |
| | 8-852.07 | 8-852.07 | 8-852.07 | 8-852.07 | 8-852.07 | 8-852.07 |
| | 8-852.08 | 8-852.08 | 8-852.08 | 8-852.08 | 8-852.08 | 8-852.08 |
| | 8-852.09 | 8-852.09 | 8-852.09 | 8-852.09 | 8-852.09 | 8-852.09 |
| | 8-852.0a | 8-852.0a | 8-852.0a | 8-852.0a | 8-852.0a | 8-852.0a |

## eReferences


e1. van Walraven C, Austin PC, Jennings A, Quan H, Forster AJ: A modification of the Elixhauser comorbidity measures into a point system for hospital death using administrative data. Med Care 2009; 47: 626-33.

e2. Quan H, Sundararajan V, Halfon P, et al.: Coding algorithms for defining comorbidities in ICD-9-CM and ICD-10 administrative data. Med Care 2005; 43: 1130-9.

e3. Quan H, Li B, Couris CM, et al.: Updating and validating the Charlson comorbidity index and score for risk adjustment in hospital discharge abstracts using data from 6 countries. Am J Epidemiol 2011; 173: 676-82.
